# The role of the type VI secretion system in the stress resistance of plant-associated bacteria

**DOI:** 10.1007/s44154-024-00151-3

**Published:** 2024-02-20

**Authors:** Rui Yin, Juanli Cheng, Jinshui Lin

**Affiliations:** Shaanxi Key Laboratory of Chinese Jujube, College of Life Sciences, Yan’an University, Yan’an, 716000 Shaanxi China

**Keywords:** Type VI secretion system, Plant-associated bacteria, Biotic stress, Abiotic stress, Environmental adaptability

## Abstract

The type VI secretion system (T6SS) is a powerful bacterial molecular weapon that can inject effector proteins into prokaryotic or eukaryotic cells, thereby participating in the competition between bacteria and improving bacterial environmental adaptability. Although most current studies of the T6SS have focused on animal bacteria, this system is also significant for the adaptation of plant-associated bacteria. This paper briefly introduces the structure and biological functions of the T6SS. We summarize the role of plant-associated bacterial T6SS in adaptability to host plants and the external environment, including resistance to biotic stresses such as host defenses and competition from other bacteria. We review the role of the T6SS in response to abiotic factors such as acid stress, oxidation stress, and osmotic stress. This review provides an important reference for exploring the functions of the T6SS in plant-associated bacteria. In addition, characterizing these anti-stress functions of the T6SS may provide new pathways toward eliminating plant pathogens and controlling agricultural losses.

## Introduction

The relationships among bacteria, their hosts, and other microbes in the environment (Stubbendieck et al. 2016) determine the conditions for bacterial adaptation to the environment determine the conditions for bacterial adaptation to the environment. Bacteria must protect themselves from adverse environmental conditions and host immune system attacks (Tang et al. 2022). Bacteria have evolved diverse survival mechanisms in response to selection pressures, including the use of protein secretion systems to combat environmental stresses (Kempnich and Sison-Mangus. 2020). The bacterial type VI secretion system (T6SS) is a contact-dependent injection mechanism that usually targets bacterial competitors (Mougous et al. 2006; Pukatzki et al. 2006; Le Goff et al. 2022) and eukaryotic hosts (Hood et al. 2010; Liu et al. 2023).

The T6SS system was originally found in plant-associated bacteria, where it has many important functions, including helping the bacteria cope with stresses from other bacteria or the plant hosts (Bladergroen et al. 2003; Gallegos-Monterrosa and Coulthurst. 2021; Wang et al. 2021b). In this paper, we review the structural assembly of T6SS and the involvement of T6SS in the stress resistance of plant-associated bacteria. In addition, the role of T6SS in the resistance of plant-associated bacteria to plant hosts is discussed.

## T6SS

**Fig. 1:**
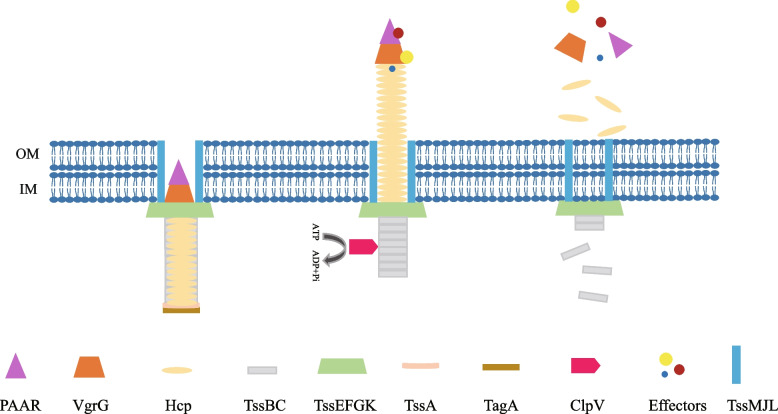
Schematic diagram of assembly and secretion of the T6SS. The T6SS is assembled and activated. After secreting the effector protein, the outer sheath of TssB-TssC is depolymerized and the T6SS is disassembled

The T6SS is a syringe structure similar to a contractile phage tail that usually consists of 13 conserved components, named TssA to TssM, forming a structure that is capable of secreting proteins (effectors) into the target cell or the extracellular environment (Fig. 1) (Cherrak et al. 2019). The structure of T6SS consists of the transmembrane complex, the baseplate complex, and the tail-tube complex (Ho et al. 2014; Basler 2015; Nguyen et al. 2017; Taylor et al. 2018). The transmembrane complex is formed by several transmembrane proteins. Most bacterial T6SS gene clusters encode three membrane proteins: TssJ, TssL, and TssM. TssL and TssM are anchored in the inner membrane, while TssJ, as an outer membrane-associated lipoprotein, exists in the periplasm (Aschtgen et al. 2008; Felisberto-Rodrigues et al. 2011). The baseplate complex is wedge-shaped and is composed of TssK, TssF, TssG, and TssE. There is a close relationship between the baseplate complex and other structures, and its function is to anchor the tail-tube complex to the transmembrane complex to initiate T6SS assembly (Zoued et al. 2016; Cherrak et al. 2018). The tail-tube complex is built on the baseplate complex and can be assembled in a short time, forming a tubular structure in the cytoplasm that includes the Hcp (TssD) inner tube, the TssB-TssC outer sheath, and the VgrG (TssI)-PAAR tip complex located at the top of the sheath (Shneider et al. 2013; Ho et al. 2014). The assembly of the T6SS is thought to begin with the assembly of the transmembrane complex, followed by the recruitment of the baseplate complex and the extension of the sheath (Wang et al. 2019). First, the outer membrane lipoprotein TssJ recruits the inner membrane proteins TssM and TssL to form a transmembrane complex (Cherrak et al. 2018). Next, the inner tube and sheath of the tail-tube complex are coordinated for assembly, where the Hcp protein and TssBC are recruited to the baseplate complex and are stacked continuously, while the TssBC sheath is rapidly polymerized to establish a tubular structure about one micron long (Basler et al. 2012). The contraction of the sheath pushes the inner tube of Hcp, the VgrG-PAAR complex, and the carried effector protein to the target cell, thereby completing the transport of the effector protein (Ruhe et al. 2020). After the delivery of the effector protein, ClpV (TssH) AAA+ ATPase binds to the contracted TssBC outer sheath for disassembly into subunits that can be recycled for the next T6SS assembly (Pietrosiuk et al. 2011; Kapitein et al. 2013).

The T6SS system was first discovered and studied in the plant-associated bacterium *Rhizobium leguminosarum* that affects pea nodulation and nitrogen fixation (Bladergroen et al. 2003). However, with the deepening of research, the T6SS has been demonstrated to have diverse functions (Lin et al. 2021), including anti-bacterial (Trunk et al. 2018) and anti-host activities (Coulthurst. 2019), mediating the uptake of metal ions (Lin et al. 2017; Han et al. 2019), resistance to environmental stress (Si et al. 2017a; Wan et al. 2017), inhibition of bacterial infection in the host (Bendor et al. 2015), and regulating the formation of bacterial biofilms (Lin et al. 2015; Chen et al. 2020). At present, the literature on T6SS primarily focuses on animal bacteria, while there are fewer studies on the function of plant-associated bacterial T6SS (Wang et al. 2023).

## T6SS confers resistance to biotic stresses

**Fig. 2:**
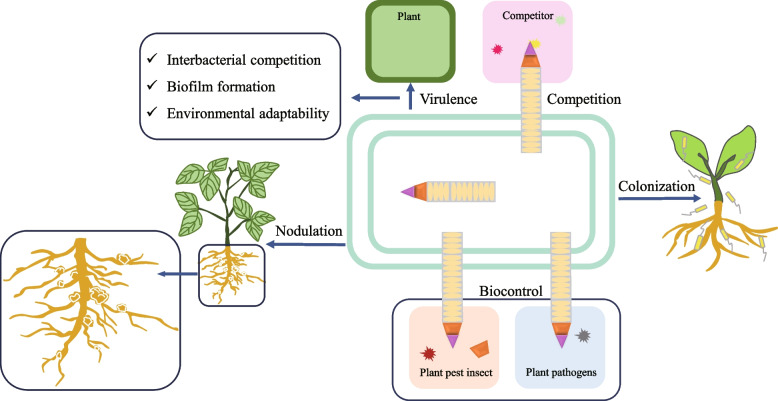
A model of T6SS involved in the biotic stress resistance of plant-associated bacteria. The T6SS participates in the competition with plant pathogenic bacteria by secreting effector proteins. The T6SS affects the virulence of plant pathogens by regulating the inter-bacterial competition, biofilm formation, and environmental adaptability. The T6SS can regulate the colonization of plant pathogens and affect the symbiosis of *Rhizobium* and host by affecting nodulation

The T6SS exists in about 25% of gram-negative bacteria (Boyer et al. 2009; Bock et al. 2017), including a large number of plant-associated bacteria (Bernal et al. 2018). Plant-associated bacteria in the environment are a diverse group that includes plant-beneficial bacteria and plant pathogens (Cui et al. 2015). The T6SS functions in the resistance of plant-related bacteria to biotic stresses such as plant pathogens and adverse environmental conditions (Fig. 2; Table 1) (Turner et al. 2013; El-Saadony et al. 2022).

**Table 1:** The role of the T6SS in the resistance of plant-associated bacteria to biotic stress

Function of T6SS in coping with biotic stresses	Organism	References
Competes with plant pathogens	*A. tumefaciens* C58, *P. putida* KT2440, P. fluorescens F113, P. sabiae LMG24235	(Ma et al. 2014), (Bernal et al. 2017), (Durán et al. 2021), (Hug et al. 2023)
Resists plant pathogens and plant pests	P. fluorescens MFE01, P. putida KT2440, P. protegens CHA0, P. chlororaphis 30–84	(Decoin et al. 2014; Bourigault et al. 2023), (Bernal et al. 2017), (Vacheron et al. 2019), (Boak et al. 2022)
Affects the virulence of plant pathogens	P. syringae pv. actinidiae M228, B. glumae BGR1, X. phaseoli pv. manihotis CIO151	(Wang et al. 2021b), (Kim et al. 2020), (Montenegro Benavides et al. 2021)
Regulates the colonization of plant pathogens	Enterobacter sp. J49, Kosakonia sp. KO348, P. fluorescens F113, Azoarcus sp. BH72	(Lucero et al. 2022), (Mosquito et al. 2022), (Durán et al. 2021), (Shidore et al. 2012)
Influences the symbiosis of *Rhizobium* and hosts	R. leguminosarum RBL5523, R. etli Mim1, P. phymatum STM815, Bradyrhizobium sp. LmicA16	(Bladergroen et al. 2003), (Salinero-Lanzarote et al. 2019), (Hug et al. 2021), (Tighilt et al. 2022)

### Competition with plant pathogens

The T6SS plays an important role in the competition among bacteria (Santoriello and Pukatzki. 2023; Zhang et al. 2023). Most plant-associated bacteria that possess the T6SS have strong viability when they coexist with other bacteria (Bernal et al. 2018; Ripcke et al. 2023). The study of plant-associated bacterial T6SS shows that this competitive advantage is achieved by injecting toxic effector proteins into rival bacteria via the T6SS to kill or inhibit their growth (Whitney et al. 2013; Tang et al. 2018).

*Agrobacterium tumefaciens*, a soil bacterial species that triggers plant tumorigenesis (Huang et al. 2021), employs the T6SS to gain a competitive advantage. In an experiment on intra-plant infection, *A. tumefaciens* secreted the effector Tde that possesses anti-bacterial DNA enzyme activity via the T6SS, giving the bacteria an advantage in the competition between homologous cells and *Pseudomonas aeruginosa*. At the same time, the bactericidal effect of this effector protein can be counteracted by the cognate immunity protein Tdi in *A. tumefaciens* (Ma et al. 2014). The conservation of toxin effector protein–immune protein (EI) pairs in T6SS-dependent bacteria highlights the use of the T6SS as a widespread antibacterial weapon that is beneficial for niche colonization (Yang et al. 2018). Moreover, in addition to T6SS-related EI pairs that can affect the competitiveness of *A. tumefaciens*, recent studies have shown that genetic characteristics also have a significant impact on the antagonistic interactions between bacteria, thus opening new avenues for the study of the ecological role of competition among bacteria (Wu et al. 2019). In addition to plant pathogenic bacteria, a T6SS-mediated competitive advantage also exists in some non-pathogenic plant bacteria such as *Pseudomonas putida* (Durán et al. 2021). For example, the Tke2 protein in *P. putida* has been confirmed to be an effector protein secreted by the T6SS that belongs to the HNH nuclease superfamily and exhibits nuclease activity. This protein is dependent on K1-T6SS secretion and has an inhibitory effect on the growth of *Escherichia coli* when heterologously expressed in *E. coli* (Bernal et al. 2017). *P. putida* is thought to be able to outcompete plant pathogenic bacteria such as *Xanthomonas campestris* by the action of the T6SS. Although this phenomenon has been observed both *in vitro* and in plants, whether this depends on the effector proteins identified above remains to be verified (Bernal et al. 2017). As a plant-beneficial bacterium, *Rhizobium* can produce nutrients to promote plant growth and increase plant yield (Yang et al. 2022a). Recent studies have found that the T6SS also plays a role in the intracellular competition of *Rhizobium* and the competition between *Rhizobium* and other plant pathogens*.* A recent study identified a T6SS-dependent effector, Re78, and an immunity protein, Re79, in *Rhizobium etli* Mim1. When wild strains were co-cultured with *re78/re79* mutants, the mutants were less competitive, indicating that Re78/Re79 were related to bacterial competition in *Rhizobium etli* Mim1 (De Sousa et al. 2023). There are two sets of T6SSs (T6SS-1 and T6SS-3) in another rhizobial strain, *Paraburkholderia sabiae*. In a competition experiment with several common plant pathogens (*Pseudomonas syringae* DC3000, *Pectobacterium carotovorum* LMG2404, and *Ralstonia solanacearum* DSM9544), the mutants in which the core gene *tssC* of T6SS-1 was mutated showed lower competitiveness compared with the wild-type strain (Hug et al. 2023), indicating that the T6SS played an important role in the competition between *P. sabiae* and other plant pathogens.

In conclusion, many plant-associated bacteria contain the T6SS, and they employ the T6SS to secrete toxic effector proteins to compete with other bacteria, allowing them to gain sufficient growth advantages in the complex bacterial environment. Although the effector proteins involved in interbacterial competition have not been identified in some bacteria, phenotypic analysis has shown that the T6SS confers advantages in competition (Boak et al. 2022).

### Resistance to plant pathogens and plant pests

Crop diseases are long-term problems that cause serious economic losses and affect the sustainable supply of food (Savary and Willocquet. 2020). While chemical pesticides can prevent crop diseases, the excessive use of pesticides is harmful to both human and environmental health (Nanjani et al. 2022). Microbial agents have been recognized as feasible and environmentally friendly pesticide alternatives (Zhang et al. 2016). Recent studies have found that many plant-beneficial bacteria, as biocontrol agents, possess the T6SS (Marchi et al. 2013), and most of these beneficial plant bacteria belong to the genus *Pseudomonas.* These bacteria can inhibit plant diseases and are considered an important source of biocontrol agents (Abo-Elyousr et al. 2021; Al-Karablieh et al. 2022; Gu et al. 2022).

There is a set of T6SS in *Pseudomonas fluorescens* strain MFE01 that exerts antibacterial activity against many common rhizosphere bacteria (Decoin et al. 2014). MFE01 can inhibit *Pectobacterium atrosepticums* and resist potato tuber soft rot, while mutant T6SS strains lose the inhibitory effect on *P. atrosepticums* (Decoin et al. 2014). A recent study found that this inhibition was due to the fact that T6SS in MFE01 can secrete an effector protein Tae3^Pf^ with amidase activity that has antibacterial activity and can thus protect potato tubers from *P. atrosepticums* infection (Bourigault et al. 2023). Therefore, T6SS in *P. fluorescens* MFE01 can inhibit the plant pathogen *P. atrosepticums* and indirectly protect potato tubers from plant diseases. In addition, *P. putida* is a saprophytic soil bacterium that can colonize the roots of crop plants; it is also one of the most characteristic biocontrol plant growth-promoting bacteria (Abo-Elyousr et al. 2021). There are three kinds of T6SS (K1-T6SS, K2-T6SS, and K3-T6SS) in *P. putida* KT2440, of which K1-T6SS is primarily used against other bacteria, including many plant pathogens (Bernal et al. 2017). *P. putida* KT2440 was used with *X. campestris* to infect *Nicotiana benthamiana* leaves, and green fluorescent protein-labeled *X. campestris* was used for in-situ colonization monitoring. Compared with the wild-type KT2440 strain, the rate of leaf necrosis and the number of *X. campestris* were increased when the mutant T6SS strain and green fluorescent protein-labeled *X. campestris* co-infected *N. benthamiana* leaves (Bernal et al. 2017). These results suggest that the protective effect of *P. putida* in plant leaves is due to the decreased survival rate of *X. campestris* in leaves, and this protection depends on the T6SS. The genome of *Azospirillum brasilense* contains two complete sets of genes encoding T6SS, and studies have shown that the T6SS of *A. brasilense* exhibits antagonistic activity *in vitro* against many plant pathogens, including *Agrobacterium*, *Pectobacterium*, *Dickeya*, and *Ralstonia* species; this allows the T6SS of *A. brasilense* to provide bio-control protection against microalgae and plants against bacterial pathogens (Cassan et al. 2021).

In addition to pathogenic *Pseudomonas*, other *Pseudomonas* bacteria also employ the T6SS to resist plant pests and eukaryote bacterivores, thereby demonstrating effective biological control. The T6SS in *Pseudomonas protegens* strain CHA0 functions in insect invasion and pathogenicity. Using larvae of the cabbage butterfly *Pieris brassicaea* as a plant-feeding insect model, the T6SS was demonstrated to be able to kill insects following oral infection. This killing effect was achieved by the T6SS and its secreted effector proteins through promoting the colonization of bacteria in the insect gut and the resulting competition with commensal gut bacteria (Vacheron et al. 2019). This was the first instance in which plant-associated bacteria in the environment were shown to use the T6SS to invade plant pests and replace intestinal endophytic bacteria. This observation was consistent with the previously reported phenomenon that both *Salmonella typhimurium* and *Vibrio cholerae* could use the T6SS to invade the host gut (Sana et al. 2016; Logan et al. 2018). The biological control effect of T6SS was also observed in *Pseudomonas chlororaphis*. *Pseudomonas chlororaphis* subsp. *aureofaciens* 30–84 is a plant growth-promoting bacterium that colonizes plant roots (Boak et al. 2022) and encodes two sets of T6SSs (T6SS-1 and T6SS-2). The existence of an arbitrary set of T6SSs in strain 30–84 can also affect the predation behavior of *Tetrahymena thermophila*, *Dictyostelium discoideum*, and *Caenorhabditis elegans*. The T6SS of strain 30–84 causes a stress response in predators, thereby protecting *P. chlororaphis* subsp. *aureofaciens* 30–84 from predation (Boak et al. 2022).

In conclusion, beneficial bacteria in plants can achieve biological control by employing the T6SS to inhibit plant pathogens and eukaryote bacterivores. These examples provide an important theoretical basis for plant-beneficial bacteria as biological control agents and suggest additional pathways for the development of new pathogenic bacteria and pest control strategies for crops, thus having significance in agricultural production.

### Effects on the virulence of plant pathogens

The virulence of many plant-pathogenic bacteria is mediated by the type III secretion system (T3SS)(Meline et al. 2019; Yan et al. 2022). The T3SS can directly translocate toxic effector proteins to plant host cells (Jayaraman et al. 2020; Qiu et al. 2023). Although there are currently no reports on the direct injection of T6SS effector proteins into plant cells, many studies have shown that the loss of the T6SS reduces the virulence of plant pathogenic bacteria.

In *Pseudomonas syringae* pv. *actinidiae* M228, when the core genes *tssM* and *tssJ* of T6SS were deleted, the pathogenicity to kiwifruit branches decreased significantly, and phenotypes involving factors such as the bacterial competition ability, biofilm formation, hydrogen peroxide tolerance, and proteolytic activity were weakened (Wang et al. 2021b). This suggests that the T6SS plays an important role in pathogenic bacteria, probably via effects on bacterial competition, biofilm formation, and environmental adaptability (Wang et al. 2021b). The plant pathogen *Burkholderia glumae* BGR1, which causes bacterial panicle blight in rice, contains four T6SS gene clusters (T6SS group_1, T6SS group_2, T6SS group _ 4, and T6SS group_5). The virulence of *tssD4* and *tssD5* mutants to rice was decreased, indicating that T6SS group_4 and group_5, as eukaryotic targeting systems, are involved in the pathogenicity of bacteria to rice (Kim et al. 2020). *Xanthomonas phaseoli* pv. *manihotis* is the causal agent of cassava bacterial blight, an economically important disease in Africa and South America that causes losses that may reach 100% after three cassava production cycles. This local and systemic pathogen induces a wide combination of symptoms, including angular leaf spots, blight, wilting, dieback, gum exudation, and vascular necrosis (Montenegro Benavides et al. 2021). In one study, the T6SS core genes *vgrG*, *clpV*, *tssM*, and *hcp* in *Xpm* strain CIO151 were mutated, and the mutants were used to infect susceptible cassava plants. After 15 days of inoculation, the plant disease was alleviated compared with plants infected with wild-type bacteria, and the phenotype of the mutants was restored after genetic complementarity. The results suggest that these genes are necessary for the CIO151 infection of cassava plants, i.e., the T6SS mediates the pathogenicity of CIO151 to cassava plants (Montenegro Benavides et al. 2021).

The way in which the T6SS mediates bacterial pathogenic effects on plants is still unclear. It may be that bacteria use the T6SS to directly transfer toxic effector proteins into plant cells to produce symptoms. Alternately, the T6SS may indirectly affect virulence when the bacteria use the T6SS to gain a growth advantage in competition with other microbiota during plant colonization. A recent study performed transcriptome analysis of *tssM* mutants at the core of the type VI secretion system of *Xanthomonas perforans* and found that the T6SS affected the expression of global regulators such as *csrA*, *rpoN*, and *pho*, trigger signal cascade responses, and coordinate the expression of a series of virulence factors, stress response genes, and metabolic genes (Ramamoorthy et al. 2023). Therefore, the TssM of T6SS may be directly or indirectly involved in controlling the virulence of *X. perforans*. As such, the exact mechanism of the relationship between plant pathogenic bacteria T6SS and plant diseases remains to be clarified.

### Regulation of the colonization of plant pathogens

Studies have shown that the T6SS plays an important role in the colonization of plant-associated bacteria (Lucero et al. 2022). Plant growth-promoting bacteria function through several mechanisms, including nitrogen fixation, the solubilization of insoluble soil phosphates, phytohormone production, and the prevention of diseases caused by pathogens (Ji et al. 2022). The T6SS of some bacteria can also inhibit the colonization of other bacteria on plants, thereby affecting plant growth.

A recent study (Lucero et al. 2022) identified strain J49 of *Enterobacter* sp. in peanuts as a strain that can colonize legumes and is an efficient phosphate solubilizer. The deletion mutation of the T6SS structural gene *hcp* in J49 significantly decreased the epiphytic and endophytic colonization rates of J49 in plant roots and aerial tissues, and the indexes related to plant colonization, including biofilm formation and polygalacturonase production, were lower than those of the wild-type strain (Lucero et al. 2022). The lack of T6SS function significantly weakened the colonization ability of J49 in legumes. In addition, a similar phenomenon was observed in *Kosakonia* sp. KO348 and *P. fluorescens* F113, in which mutation of the T6SS resulted in a phenotype with significantly decreased root colonization ability (Durán et al. 2021; Mosquito et al. 2022).

In contrast, the T6SS also appears to inhibit bacterial colonization in plants (Shidore et al. 2012). Shidore et al. found that in *Azoarcus* sp. BH72, when the same amount of wild-type strain BH72 and T6SS mutant BH*azo3888* infected rice seedlings, the mutants showed stronger root colonization ability (Shidore et al. 2012). The same phenomenon was also observed in *Acidovorax citrulli*. Compared with the wild-type strain Aac5, the colony number of the strain with the mutant T6SS core gene *hcp* increased significantly after infecting watermelon cotyledons for 72 hours (Fei et al. 2022), indicating that T6SS was related to the colonization of *A. citrulli* (Kan et al. 2023).

In short, T6SS has two distinct effects on promoting and inhibiting the colonization of plant-associated bacteria in hosts. Therefore, we speculate that the inhibitory effect of T6SS on plant bacterial colonization may be related to the effector proteins secreted by the T6SS. Effector proteins secreted by the plant-associated bacterium T6SS trigger the local defense response of plants to kill wild-type strains. Since the T6SS mutant cannot secrete the effector protein, it will not trigger the local defense response of the plant, and thus the bacteria can effectively colonize the host plant (Shidore et al. 2012). However, this only explains how the T6SS inhibits bacterial colonization of plants, while the observation that the T6SS promotes bacterial colonization in plants may be explained by the inhibition of plant local defense responses by effector proteins. Therefore, if we can identify a T6SS effector protein that acts on plants and that has an effect on plant local defense systems, it will undoubtedly be a breakthrough in the field of T6SS research (Wang et al. 2023).

### Influences on the symbiosis of *Rhizobium* and hosts

The T6SS exists widely in the genus *Rhizobium* and functions in both the promotion and inhibition of the symbiotic relationship between *Rhizobium* and legumes (Yang et al. 2022a).

Before the *V. cholerae* T6SS was identified, a gene encoding the T6SS had been identified in legume RBL5523, where the T6SS key gene *impJ* (*tssK*) mutant obtained via the insertion mutation of the Tn5 transposon caused pea plants to form more nodules and perform nitrogen fixation more effectively. This indicated that the T6SS could inhibit nodulation and nitrogen fixation in peas by inhibiting the symbiosis between *Rhizobium* and legumes (Bladergroen et al. 2003).

However, later studies have been more inclined to support the promoting effect of the T6SS on the symbiosis between *Rhizobium* and legumes. One study found that the plant dry weight and root nodule size produced by the mutation of the T6SS structural gene in Mim1 were lower than those of the wild-type strain. This was the first demonstration that the T6SS plays a promoting role in the symbiosis between *Rhizobium* and legumes (Salinero-Lanzarote et al. 2019). Similar phenomena have subsequently been reported in the rhizobia of many leguminous plants. For example, *Paraburkholderia phymatum* STM815 can nodulate a broad range of legumes, including the agriculturally important *Phaseolus vulgaris* (common bean) (Bellés-Sancho et al. 2023). *P. phymatum* harbors two T6SSs (T6SS-b and T6SS-3) in its genome. Differential gene expression analysis showed that T6SS-b could be activated in soil and the rhizosphere (Hug et al. 2021). Although no visible symbiotic phenotypes of the T6SS-b mutant were observed during the infection of germinated bean seeds, T6SS-b was expressed in the area of the root tip, suggesting that the expression of T6SS-b was caused by a component present on the tip of the root and indicating that T6SS was involved in early symbiosis with bean roots. *R. leguminosarum* LmicA16 is necessary for efficient nodulation in *Lupinus micranthus* and *Lupinus angustifolius*. After the mutation of the T6SS core structural gene *vgrG* in LmicA16, the mutant produced fewer nodules and smaller plants compared with the wild-type strain, and its competitiveness was weaker when co-inoculated with the wild-type strain. In addition, Tsb1 is a protein whose coding function is unknown but that contains a methyltransferase domain. The *tsb1* mutant showed a phenotype between that of the wild type and the *vgrG* mutant. The effect of the *tsb1* mutation suggests that the Tsb1 protein could be a possible T6SS effector that has a positive effect on the symbiosis between LmicA16 and *Lupinus* spp. (Tighilt et al. 2022).

Although no T6SS effector for the host cells of leguminous plants has been described, the beneficial effect of T6SS in symbiosis may be related to the attenuation of the defense response of the compatible host plant, as described by T3SS-dependent rhizobial effectors as effector-triggered susceptibility (Berrabah et al. 2019). This information may be significant for both ecological and agricultural research.

In conclusion, plant-associated bacteria improve their environmental adaptability and that of their plant hosts by employing the T6SS to compete with plant pathogens, inhibit plant pests, and influence colonization and symbiosis with their plant hosts. These examples provide an important theoretical basis concerning how some plant-associated bacteria resist biotic stresses in the environment, and thus provide further possibilities for developing new pathogen and pest control strategies to improve crop health.

## T6SS confers resistance to abiotic stresses

**Fig. 3:**
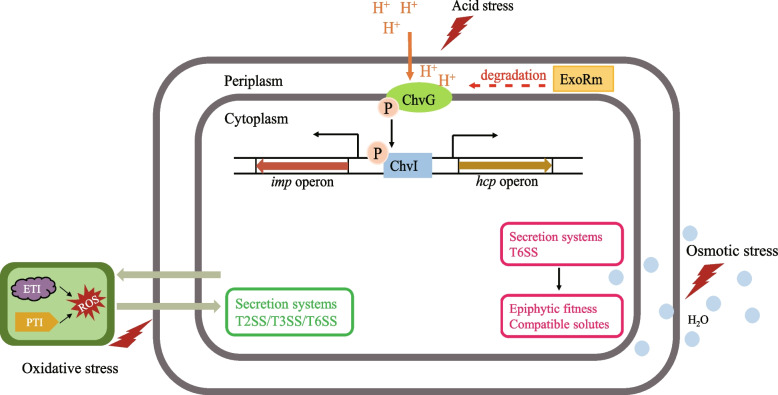
A model of the T6SS involved in the abiotic stress resistance of plant-associated bacteria. Under acid stress, acid-induced degradation of ExoRm may derepress ChvG to activate the T6SS of plant-associated bacteria by phosphorylation of the ChvI response regulator. Bacterial invasion of plants triggers plant immune defenses, leading to oxidative stress and osmotic stress. The expression of related genes in the T6SS gene cluster is altered and affects plant-associated bacterial resistance to stress

Plants have complex immune response strategies to defend against most microbes (Nishad et al. 2020). The immune defense response in plants includes pattern−triggered immunity (PTI) and effector-triggered immunity (ETI) (Spoel and Dong. 2012). The plant membrane pattern recognition receptors (PRRs) recognize microbe-associated molecular patterns (MAMPs) to trigger immune responses, thereby resulting in the accumulation of reactive oxygen species (ROS) (De Lorenzo et al. 2018). In addition, when infected by pathogens, plants will also produce organic acids or cause changes in the concentration of calcium and potassium ions inside and outside the cell, thus creating an acidic rhizosphere environment and a high osmotic gradient. Therefore, in addition to resistance to biotic stresses, bacteria are also resistant to various environmental stresses (Bhattacharyya et al. 2021) as well as some abiotic stresses released by the plant hosts, including oxidative stress, acid stress, and osmotic stress (Fig. 3) (Cui et al. 2015; Przepiora et al. 2019). There is some evidence that the T6SS plays a role in affecting the resistance of plant-associated bacteria to environmental stress and thus helps the cells to survive under adverse environmental conditions.

### Acid stress

The intracellular pH plays an important role in regulating a variety of cellular processes, including cell growth, cell adhesion, signal transduction, and endocytosis (Wang et al. 2010). Bacteria infecting plant cells encounter a low pH in the apoplast (Jiang et al. 2016), and plants also release phenolic compounds as well as neutral and acidic sugars necessary to repair damaged tissue, thereby acidifying the rhizosphere to create a low pH environment that can limit the growth of pathogens (Winans. 1992; Subramoni et al. 2014). In response, pathogens have evolved multiple adaptation mechanisms, including using the T6SS in the stress response to low intracellular pH (Zhang et al. 2013; Qin et al. 2020; Yu et al. 2021).

Some plant-associated bacterial T6SSs can play a role in the intracellular low-pH stress response (Leonard et al. 2017). For example, when *A. tumefaciens* is near a suitable plant host in the rhizosphere, acidic conditions and plant-derived chemicals play important roles in activating its virulence (Winans. 1992; Yuan et al. 2008a). At the same time, after sensing the acidic features of the rhizosphere, *A. tumefaciens* exerts a conserved and signal-specific response to the plant host and adapts to the rhizosphere niche by regulating metabolism and cellular adaptation (Yuan et al. 2008a). *A. tumefaciens* can sense acidic pH through the ChvG/ChvI two-component system and activate the expression of several virulence factors, including the T6SS coding gene cluster (Yuan et al. 2008b; Subramoni et al. 2014). The periplasmic repressor ExoR negatively regulates the receptor kinase ChvG and reduces the expression and secretion activity of the T6SS. At acidic pH levels, the acid-induced degradation of periplasmic ExoR (ExoRm) may derepress ChvG to activate the T6SS through the phosphorylation of the ChvI response regulator (Wu et al. 2012). Due to the widespread existence of the ChvG/ChvI two-component system in α-Proteobacteria, the cascade regulation of the T6SS through ExoR-ChvG/ChvI may be a universal regulation mechanism in plant pathogens.

In addition to the ChvG/ChvI two-component system, there are other two-component systems capable of regulating the T6SS to help bacteria adapt to an acidic environment. OmpR is a well-characterized factor from the EnvZ/OmpR two-component system that regulates the expression of genes in response to changes in pH in the environment (Gerken et al. 2009). In *Yersinia pseudotuberculosis*, T6SS4 regulated by OmpR is essential for the bacteria to survive in acidic conditions, and OmpR can activate the T6SS to pump H^+^ out of cells to maintain the intracellular dynamic balance of pH. This may be a general strategy for pathogens to resist acidic conditions (Zhang et al. 2013). Alternatively, H3-T6SS, which is negatively regulated by OmpR in *P. aeruginosa*, and its secreted effector protein TepB, were able to aid in bacterial survival under acidic conditions (Yang et al. 2022b).

The regulatory mechanism of acid-induced T6SS is common in bacteria, and most feature two-component systems to respond to an external acidic environment. Whether acid-induced T6SS is a common regulatory mechanism in plant-associated bacteria in addition to the Exor-ChvG/ChvI cascade system is as yet unclear, as is the mechanism by which the T6SS participates in the response of plant bacteria against acid stress.

### Oxidative stress

The production of ROS plays a key role in activating plant defense mechanisms (Torres. 2010; Khan et al. 2023). Through the activation of NADPH oxidases and peroxidases caused by the perception of pathogen-associated molecular patterns (PAMPs) by plants through PRRs, ROS are produced that in turn induce PTI-dependent basal defenses against invading pathogens. Following the perception of a PAMP, the produced ROS also act as toxins that can kill the pathogens (Park et al. 2013).

However, pathogens have evolved multiple mechanisms to evade host defenses by counteracting the deleterious effects of ROS (Wang et al. 2022). The T6SS is also involved in the evasion of host defenses. For example, *Dickeya dadantii*, a pathogenic bacterium that causes soft rot in many crops, employs the T6SS to combat oxidative stress and improve its environmental adaptability. In response to H_2_O_2_ treatment, *D. dadantii* expressed chemotactic genes to escape from the locally toxic environment and invade the plant intercellular space (Jiang et al. 2016). At the same time, the expression of several genes related to the type II, V, and VI secretion systems was downregulated (Jiang et al. 2016), indicating that the pathogen prioritized the allocation of its energy to survive rather than secrete late virulence factors. This phenomenon also shows that the T6SS of the plant-associated bacterium *D. dadantii* is involved in the process of resisting external oxidative stress through a special mechanism (Reverchon et al. 2016).

Unlike the T6SS, the use of the T3SS by plant pathogens to combat oxidative stress has been investigated, and studies have revealed antagonistic mechanisms. For example, the interaction between *Ustilago maydis* effector ROS burst interfering protein 1 (Rip1) and maize susceptibility factor lipoxygenase 3 (Zmlox3) results in the suppression of the ROS burst in several subcellular compartments of plant cells (Saado et al. 2022). Another way to resist oxidative stress is through the interaction between the effector protein and the target protein in the host. *P. syringae* effector HopAO1 targets lipooligosaccharide-specific reduced elicitation (LORE), a G-type lectin receptor-like kinase, to inhibit ROS bursts in *Arabidopsis* (Luo et al. 2020). Mechanistic studies of T3SS-secreted effector proteins against host oxidative stress also provide insights into the function of the T6SS. Although the mechanism of the T6SS in plant pathogens against oxidative stress in the environment is currently unclear, the T6SS in other bacteria is employed to resist oxidative stress by secreting effector proteins. For example, when Enterohemorrhagic *E. coli* (EHEC) colonizes the human intestine, macrophages will produce ROS to inhibit the bacteria. *E. coli* has a variety of different catalases to defend against oxidative stress (Poole. 2005; Imlay. 2008). A recent study showed that the T6SS of EHEC can secrete an effector protein KatN with catalase activity that can reduce the ROS level of host cells, thereby promoting the survival of EHEC in phagocytes (Wan et al. 2017). In addition, T6SS1 in *Cupriavidus necator*, which is regulated by the transcription factor Fur, secretes the lipopolysaccharide-binding effector TeoL to construct outer membrane vesicles in response to oxidative stress (Li et al. 2022). Therefore, in our future research, we will search for T6SS effector proteins examine their effects on host ROS levels, and further explore novel functions of T6SS in helping plant pathogens cope with oxidative stress.

### Osmotic stress

The defense response of plants after infection promotes the influx of calcium ions to plant cells and the massive efflux of potassium ions in the apoplasts, resulting in osmotic pressure changes and cytoplasmic wall separation (Garcia-Brugger et al. 2006). Plant pathogens thus face osmotic stress when infecting plant cells. The tolerance to osmotic stress varies among bacteria. Differences in osmotic tolerance may be related to diverse adaptation strategies involving differences in osmotic adaptation mechanisms. These differences will affect the relative fitness of strains (Freeman et al. 2013). Studies have shown that the T6SS can improve bacterial osmotic adaptability (Gueguen et al. 2013; Guan et al. 2015), and this phenomenon also exists in plant bacteria.

Research has demonstrated that *P. syringae* B728a has strong osmotic tolerance. A whole-transcriptome analysis of B728a showed that 10 genes in the T6SS-Ι cluster, including *clpV* and *tssM*, are induced when the osmotic pressure increases (Freeman et al. 2013). According to the role of T6SS in antagonistic bacterial cell–cell interactions during protein transfer, the osmotic induction of the core T6SS machinery may be relevant to the interaction of B728a with other epiphytic organisms and may contribute to epiphytic fitness by antagonizing other microbes (Basler et al. 2013). In addition, the presence of *tssM* in *X. perforans* provides osmotic stress adaptation, facilitating the epiphytic colonization and growth of the pathogens (Liyanapathiranage et al. 2022). In the presence of a certain concentration of NaCl, compared with the *tssM* mutant, the wild-type strain *X. perforans* AL65 showed higher growth (Liyanapathiranage et al. 2022). This indicates that T6SS plays a role in adaptation to osmotic stress in the plant-associated bacterium *X. perforans*. In summary, T6SS is involved in the resistance of some plant-associated bacteria to osmotic stress. However, the ecological benefits of osmotic stress as a signal for T6SS induction are unclear.

The accumulation of compatible solutes is a strategy for bacteria to defend against high osmotic pressures in the environment (Kempf. 1998; Zeidler and Müller. 2019). The accumulation of specific compatible solutes in the bacterial cytoplasm also contributes to in-plant fitness in a strain-specific manner (Wang et al. 2021a). In addition, the responses of cells to osmotic pressure include potassium ion import. For example, bacteria use the two-component system KdpDE to regulate the transport of K^+^ by different proteins to maintain osmotic balance (Epstein. 2016; Schramke et al. 2016). Bacteria use the T6SS to transport metal ions (Lin et al. 2017; Si et al. 2017b; Han et al. 2019), and thus the T6SS in plant bacteria may also achieve osmotic adaptability by regulating the concentrations of intracellular and extracellular calcium and potassium ions. Future studies can take this as a starting point to investigate the mechanism by which the T6SS aids plant bacteria in resisting osmotic pressure.

In conclusion, the expression of the T6SS in plant-associated bacteria responds to external abiotic factors such as acid stress, oxidative stress, and osmotic stress. However, whether abiotic stress-induced T6SS is a common regulatory mechanism in plant-associated bacteria as well as the biological significance and application value of such regulation are important issues for future research.

## Concluding remarks

The T6SS has been extensively studied as a versatile bacterial component. The T6SS was first described in *V. cholerae*, and subsequent research has largely focused on animal bacteria (Alteri and Mobley. 2016; Hernandez et al. 2020). However, the T6SS is also an extensive and very important system in plant-associated bacteria. This review summarizes the diverse functions of the T6SS in some common plant-associated bacteria, including the important role of the T6SS in resistance to biotic and abiotic stresses. In the resistance to biotic stress, the T6SS helps plant-associated bacteria improve their environmental adaptability and that of their plant hosts by competing with plant pathogens, inhibiting plant pests, and affecting colonization and symbiosis with plant hosts. However, in contrast to animal bacteria, T6SS effectors that can be directly secreted into plant cells have not yet been found, and the phenotypes of T6SSs that help plant-associated bacteria resist biological stresses have not been characterized. This is a research direction that requires further investigation in future studies.

There are few studies on plant-associated bacteria using the T6SS to resist abiotic stresses. The expression of T6SS-related genes has been shown to be altered under abiotic stress conditions. Although it has been speculated that this is due to the upregulation and downregulation of genes triggered by plant bacteria to resist environmental stress, the specific regulatory mechanisms remain unclear. The T6SS is regulated by multiple complex two-component systems in the cell, and some two-component systems can modulate the expression of the T6SS in response to abiotic stresses on plants, for example, the ExoR-ChvG/ChvI systems of *A. tumefaciens* that can activate the T6SS under acidic conditions. Further studies can target the regulation of the T6SS by abiotic stress-related intracellular components, explore the role of the T6SS in plant-associated bacterial resistance to abiotic stress, and expand the known functions of the T6SS in plant-associated bacterial stress resistance.

Plant-associated bacteria have been demonstrated to use the T6SS to compete with plant pathogens and adapt to abiotic stresses. This research provides a theoretical basis for improving the environmental adaptability of plant-associated bacteria and plant hosts. Therefore, it is crucial to explore the molecular mechanisms involving the T6SS in the interactions between plant-associated bacteria and their plant hosts. This may become an important direction for T6SS research, and it has potential application to the reduction of agricultural losses.

## Data Availability

Not applicable.

## Abbreviations

T6SS: Type VI secretion system
EI: Effector protein-immune protein
T3SS: Type III secretion system
ExoRm: Periplasmic ExoR
PTI: Pattern−triggered immunity
ETI: Effector-triggered immunity
PRRs: Pattern recognition receptors
MAMPs: Microbe-associated molecular patterns
ROS: Reactive oxygen species
PAMPs: Pathogen-associated molecular patterns
Rip1: ROS burst interfering protein 1
Zmlox3: Maize susceptibility factor lipoxygenase 3
LORE: Lipooligosaccharide-specific reduced elicitation
